# Real-World Effectiveness of Golimumab in Ulcerative Colitis: A Pooled Analysis from the Prospective UMBRELLA-IBD Registry in Germany

**DOI:** 10.3390/jcm14207347

**Published:** 2025-10-17

**Authors:** Arne Bokemeyer, Thomas Wenske, Sandra Plachta-Danielzik, Bernd Bokemeyer

**Affiliations:** 1 Department for Gastroenterology, Diabetology and Palliative Care, Bonifatius Hospital Lingen, 49808 Lingen, Germany; 2 Competence Network IBD, 24103 Kiel, Germany; t.wenske@ced-service.de (T.W.); s.plachta-danielzik@kompetenznetz-darmerkrankungen.de (S.P.-D.); bernd.bokemeyer@t-online.de (B.B.); 3 Interdisciplinary Crohn Colitis Center, 32429 Minden, Germany

**Keywords:** golimumab, treatment persistence, effectiveness, inflammatory bowel disease, ulcerative colitis

## Abstract

**Background:** The efficacy and safety of the anti-TNF inhibitor golimumab in ulcerative colitis (UC) have been demonstrated in pivotal randomized controlled trials (RCTs). However, real-world data are needed to assess its effectiveness and safety in routine clinical practice, where patient populations and treatment settings are more heterogeneous. **Methods:** This pooled, retrospective–prospective cohort analysis draws on primary data from four IBD registries that contribute UC data to the UMBRELLA-IBD data warehouse: BioColitis, RUN-UC, TARGET-IBD, and VEDO-IBD. Data for each registry were collected across multiple centers under routine clinical care conditions in Germany. Eligible patients had a confirmed diagnosis of UC according to DGVS/ECCO criteria, were ≥18 years of age, and had newly initiated treatment with golimumab between 2017 and 2023. In total, 222 patients met these criteria and were included in the analysis. Statistical analyses included descriptive summaries, group comparisons, and Kaplan–Meier analysis of treatment persistence. Adverse events (AEs) and serious adverse events (SAEs) were also assessed and compared to other anti-TNF therapies from UMBRELLA-IBD. **Results:** Of the 222 patients who newly initiated golimumab, 134 had a documented month 12 visit with a documented pMayo score and were included in the modified intention-to-treat (mITT) analysis. A high proportion of the patients in the study had previously received treatment with at least two biologics (81%). In this mITT population, clinical remission was achieved in 38.1% and steroid-free clinical remission (SFCR) in 36.6% at 12 months. In the full cohort, treatment persistence at 12 months was 67.2%. Safety data on adverse events (AEs) and serious adverse events (SAEs) were reported in 14.8% and 5.8% of cases, respectively, with no significant differences compared with other anti-TNF therapies. **Conclusions:** In addition to the positive findings from the pivotal RCTs, these real-world data further support the clinical effectiveness and safety of golimumab in routine care for UC.

## 1. Introduction

Biologic therapies have substantially transformed the management of inflammatory bowel disease (IBD) in recent years. Golimumab, a fully human monoclonal antibody targeting TNF-α, is an established treatment option for patients with moderately to severely active ulcerative colitis (UC) who respond inadequately to, or are intolerant of, conventional therapies. Its efficacy and safety have been demonstrated in pivotal randomized controlled trials (PURSUIT-SC and PURSUIT-Maintenance) [1,2]. However, real-world data are needed to better characterize its effectiveness and use in routine clinical practice, where patient populations and treatment conditions are more heterogeneous.

One example is the GO-CUTE study, a prospective, non-interventional, single-arm, real-world investigation in Germany that followed 117 patients with moderate to severe UC for up to 12 months [3]. Treatment with golimumab resulted in significant improvements in work productivity, daily functioning, and health-related quality of life (HRQoL), as measured by the Work Productivity and Activity Impairment (WPAI) questionnaire and the 12-Item Short Form Survey (SF-12). Further real-world evidence comes from a 22-week prospective observational study in South Korea, which included 130 patients with UC [4]. In that cohort, 48.6% achieved clinical remission (CR) at week 22, as measured by the partial Mayo (pMayo) score, while adverse drug reactions were observed in only 4.6%. Lastly, a systematic review and meta-analysis by Olivera et al. (2019) [5] synthesized real-world evidence from eight observational studies including 822 patients, reporting a pooled CR rate of 39.2% at 24 to 54 weeks.

Although these studies provide important data, real-world evidence on golimumab remains limited, particularly from large, multicenter cohorts with systematically collected patient-level data that reflect routine care. Moreover, most real-world studies to date have reported golimumab results only within the broader class of anti-TNF therapies, without providing summary estimates specific to golimumab. The UMBRELLA-IBD data warehouse addresses these gaps by consolidating prospectively collected data from multiple German IBD registries, now encompassing more than 6500 patients. Established to enable pooled analyses of advanced therapies, it provides harmonized, high-quality longitudinal data at the individual level and allows for golimumab-specific analyses that account for key clinical effect modifiers [6].

Against this background, we conducted a pooled analysis of all UC patients from UMBRELLA-IBD who had newly initiated golimumab therapy in routine clinical care. The aim was to describe demographic and clinical characteristics, evaluate treatment response and persistence, and assess safety in a real-world setting. These findings are intended to clarify the role of golimumab in clinical practice and support evidence-based treatment decisions in UC.

## 2. Methods

### 2.1. Study Design and Data Sources

This pooled, retrospective–prospective cohort analysis is based on primary data from the four German registries that contribute UC data to the UMBRELLA-IBD data warehouse: BioColitis, RUN-UC, TARGET-IBD, and VEDO-IBD. Each registry collects data prospectively from multiple sites under routine clinical care conditions following standardized documentation protocols and quality-assured procedures, including remote and on-site monitoring. Ethical approval was obtained for each registry, and all patients provided written informed consent; therefore, this study has been performed in accordance with the ethical standards laid down in the 1964 Declaration of Helsinki and its later amendments.

BioColitis contains clinical data on 880 UC patients and enables comparisons of biologic therapies in everyday practice; previous analyses have informed the positioning of TNF inhibitors [7]. RUN-UC tracks the real-world use of ustekinumab, anti-TNF agents, and vedolizumab in 507 UC patients, reporting high treatment persistence and favorable tolerability across agents [8]. TARGET-IBD is a registry based on electronic health records, collecting prospective data on the use and safety of advanced therapies across diverse clinical settings (current recruitment status in December 2024: 647 UC patients) [9]. VEDO-IBD was established to assess vedolizumab and other biologics, providing up to two years of follow-up in 512 UC patients in Germany [10].

### 2.2. Patient Cohort and Homogeneity Analysis

**Figure 1:**
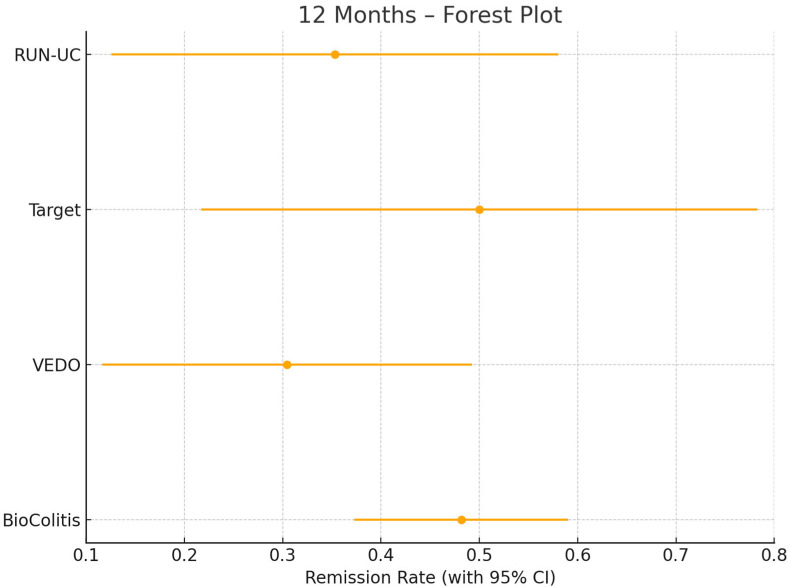
12-month clinical remission (CR) rates with 95% confidence intervals among 133 golimumab-treated patients from the four contributing registries (RUN-UC, *n* = 17; VEDO-IBD, *n* = 23; TARGET, *n* = 12; BioColitis, *n* = 81) included in the pooled analysis. The estimated clinical remission rates are indicated by dots and the lines represent the corresponding 95% confidence intervals.

For the present analysis, all patients were included who (a) had a confirmed diagnosis of UC according to the criteria of the German Society for Gastroenterology, Digestive and Metabolic Diseases (DGVS) and the European Crohn’s and Colitis Organisation (ECCO), (b) were ≥18 years of age, and (c) had newly initiated treatment with golimumab between 2017 and 2023. Patients with prior exposure to golimumab or incomplete baseline data were excluded. A total of 222 patients met the inclusion criteria. To assess the appropriateness of pooling data from the four registries, we conducted a homogeneity analysis based on 12-month CR rates calculated separately for each registry, as shown in Figure 1. Cochran’s Q test indicated no statistically significant heterogeneity among the studies (*Q* = 3.25, *df* = 3, *p* = 0.35), and an I^2^ statistic of 7.7% suggested only minimal heterogeneity, supporting the statistical appropriateness of pooling the data sets for joint analysis. This strategy for pooling individual patient data is further supported by simulation studies [11], which have shown that analyses of individual patient data (IPD) yield more valid and precise estimates than aggregate-data methods such as network meta-analyses, particularly in sparse networks or when treatment effect modifiers are relevant.

### 2.3. Baseline Data and Treatment Regimens

At baseline, the following patient characteristics were recorded: age, sex, body weight, disease duration, prior therapies (particularly previous anti-TNF exposure), endoscopic disease activity, C-reactive protein levels, and clinical disease activity (partial Mayo score; pMayo). Golimumab dosing adhered to the approved label in effect at the time of treatment initiation: Until June 2018, patients weighing < 80 kg received an initial dose of 200 mg subcutaneously at week 0, followed by 100 mg at week 2 and then 50 mg every four weeks for maintenance. Patients weighing ≥ 80 kg received 100 mg every four weeks for maintenance. Beginning in July 2018, all patients received a fixed maintenance dose of 100 mg subcutaneously every four weeks regardless of body weight.

### 2.4. Outcomes and Definitions

The main outcome was CR at month 12, defined as a partial Mayo (pMayo) score ≤ 1 with a rectal bleeding subscore of 0. The primary effectiveness analysis was a modified intention-to-treat (mITT) analysis of patients with a documented month 12 visit. Additional outcomes included steroid-free clinical remission (SFCR), treatment persistence, and CR at month 6. As there were no data available from TARGET-IBD and BioColitis at the end of the induction phase, CR at this time point was analyzed using data from VEDO-IBD and RUN-UC. Safety was evaluated based on the frequency of adverse events (AEs) and serious adverse events (SAEs) during the first 12 months of follow-up, recorded through standardized reporting protocols across registries and classified according to standard pharmacovigilance definitions.

### 2.5. Statistical Analysis

Descriptive analyses were performed to characterize the overall population and relevant subgroups. Categorical variables are reported as absolute and relative frequencies, and continuous variables as mean with standard deviation or as median with interquartile range (IQR), as appropriate. Treatment persistence over the 12-month follow-up period was evaluated using Kaplan–Meier survival analysis. Group comparisons were conducted using chi-squared tests, Fisher’s exact test, *t*-tests, or nonparametric methods depending on the distribution of the data.

Analyses were conducted using both the modified intention-to-treat (mITT) and as-observed approaches. The mITT analysis included all participants who newly initiated golimumab therapy and had a pMayo score at the respective endpoint. This approach modifies the intention-to-treat principle by classifying patients who discontinued golimumab as outcome failures. Complete information was required for medication and remission status, whereas partial missingness in other variables was tolerated under the assumption that the data were missing at random. The as-observed analysis included only patients still receiving golimumab at each time point, without imputing missing values, and thus reflects the recorded outcomes.

Differences in the frequencies of AEs and SAEs between patients treated with golimumab and those receiving other anti-TNF agents were assessed using Fisher’s exact test. A two-sided *p*-value < 0.05 was considered statistically significant. To contextualize the findings, additional descriptive comparisons were made using published data on golimumab and other anti-TNF agents. All analyses were conducted using R version 4.3.1.

## 3. Results

Baseline characteristics of the 222 included UC patients are presented in Table 1. Overall, 8.0% of the patients were biologic-naïve, 11.0% had undergone one prior biologic therapy, and 81% had received at least two prior biologic therapies. This indicates that a high proportion of the patients in the study had previously received treatment with at least two biologics, suggesting that many of the patients included were relatively treatment-resistant. The median age was 39 years, and 49.5% were female. The mean body weight was 74 kg, and 38.9% of patients weighed ≥80 kg.

**Figure 2:**
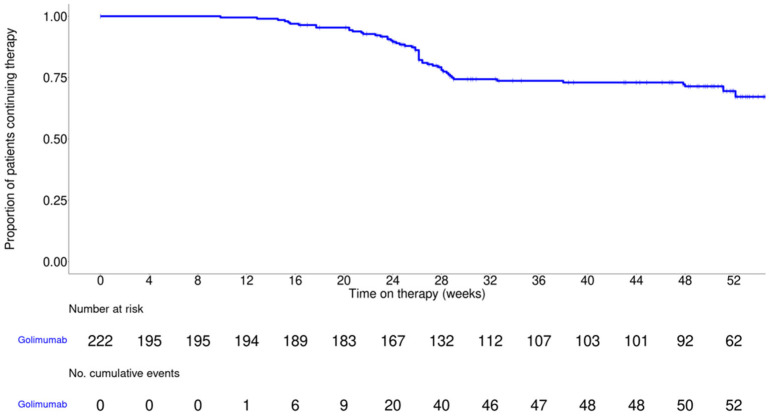
Treatment persistence with golimumab therapy over 52 weeks, as assessed by Kaplan–Meier analysis. The solid line in the graph represents the estimated probability of continuing golimumab therapy over time. The censored observations are indicated by tick marks.

Figure 2 presents 52-week treatment persistence as assessed by Kaplan–Meier analysis. At 52 weeks, 67.2% of patients remained on golimumab, indicating sustained use over time and suggesting continued clinical benefit and good tolerability in routine care.

Table 2 shows the effectiveness of golimumab in achieving CR and SFCR at months 6 and 12 based on an as-observed analysis including only those patients still receiving golimumab at each respective time point (month 6: *n* = 138; month 12: *n* = 101). At 12 months, the CR rate was 50.5%, and the SFCR rate was 48.5%.

Table 3 shows remission rates stratified by weight group (<80 kg vs. ≥80 kg) from induction through month 12 based on data from RUN-UC and VEDO-IBD, the only registries with outcomes available for post-induction and 12 months. At month 12, CR rates were numerically lower in patients weighing <80 kg (26.7%) compared with those ≥80 kg (38.5%), but none of the between-group differences at weeks 14–16, month 6, or month 12 reached statistical significance (all *p* > 0.05). Although not statistically significant, the numerical difference in CR rates between weight groups (11.8% absolute difference) may be clinically relevant and warrants investigation in larger cohorts. The study may, however, have been underpowered to detect a true difference in effectiveness between the weight groups.

**Figure 3:**
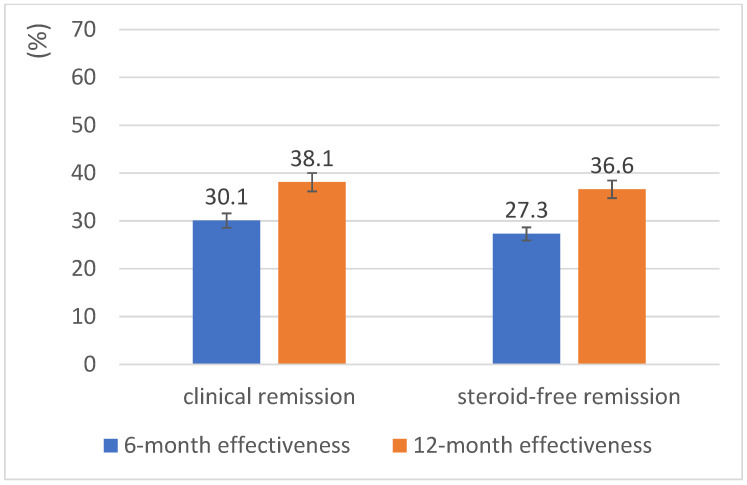
UMBRELLA-IBD pooled analysis with 6-and 12-month effectiveness of golimumab in ulcerative colitis.

Figure 3 shows the primary effectiveness analysis of golimumab in the mITT analysis of all patients with a documented month 12 visit (*n* = 134). In this analysis, a switch to another advanced therapy was considered treatment failure. Over 12 months, some patients receiving golimumab were lost to follow-up or had missing data (e.g., pMayo score). This resulted in 134 patients with golimumab data being eligible for the mITT analyses at 12 months (see Supplementary Figure S1). Although this is a limitation, data attrition is unavoidable in non-interventional, real-world studies. At month 6, the CR rate was 30.1% and the SFCR rate was 27.3%. At month 12, the CR rate had risen to 38.1%, and the SFCR rate to 36.6%.

Safety data were available for 75 patients treated with golimumab and 202 patients receiving other anti-TNF therapies from UMBRELLA-IBD, each followed for 12 months. SAEs were observed in five golimumab-treated patients (6.7%) and 11 patients (5.4%) in the anti-TNF comparator group (*p* = 0.774). Most SAEs were hospitalizations (12 patients), followed by two surgeries (partial colectomy and appendectomy), one infection (bronchopneumonia) and one episode of intestinal bleeding (secondary bleeding during a polypectomy). Overall, AEs were observed in 14 golimumab-treated patients (13.9%) and 31 patients (15.3%) in the anti-TNF group (*p* = 0.866). None of the between-group differences were statistically significant.

## 4. Discussion

In this pooled analysis of real-world data on newly initiated golimumab treatment in 222 patients with UC, a relatively high 12-month treatment persistence rate of 67.2% was observed. This suggests sustained clinical benefit and favorable tolerability under routine care conditions. Persistence, sometimes termed drug survival, is an important surrogate marker in observational research because it reflects treatment effectiveness, safety, and satisfaction among both patients and physicians [12,13].

In the mITT analysis, the CR rate at month 12 was 38.1%. This is broadly consistent with previously reported data from RCTs and real-world studies (Supplementary Figure S2). As expected, the corresponding rate in the pivotal PURSUIT trial was lower at 27.8% [2], which may reflect narrower inclusion criteria and the more controlled trial environment. Potential alternative explanations, such as physician treatment selection bias in real-world cohorts, should also be considered. In contrast, the pooled estimate from the systematic review by Olivera et al. [5] (35.9%) is more in line with the findings of the present analysis. Similarly, the 12-month remission rate reported here is also comparable to the 34.7% observed for anti-TNF agents overall in the recent RUN-UC study (2025) [8], further supporting the effectiveness of golimumab in clinical practice. Although the remission rate following maintenance therapy in the golimumab arm of the VEGA study [14] was lower at 22.2%, this difference may be attributable to the more stringent composite endpoint in VEGA, which combined CR with endoscopic mucosal improvement.

Beyond these comparisons, findings from additional real-world cohorts are consistent with our estimates. The pragmatic, phase-4 UK GO-COLITIS study reported induction and maintenance benefits on the pMayo score and patient-reported outcomes under routine care, broadly in line with trial efficacy results while reflecting everyday practice constraints [15]. Multicenter registry analyses from Italy (IG-IBD) demonstrated sustained effectiveness and acceptable safety up to two years and identified anti-TNF-naïve status as a positive predictor of response [16]. Durability is further supported by a Spanish cohort, with drug persistence of approximately 60% at 12 months, declining to around 25–30% by year 4, albeit with many patients regaining response after a dose intensification of golimumab [17]. In settings where loss of response occurs, a prospective series suggested that golimumab intensification can be effective without strict dependence on drug-level-guided thresholds, underscoring the practical usability of this agent in routine algorithms [18]. Collectively, these data support golimumab as an effective and durable option in real-world UC management, consistent with the persistence and remission rates observed in our analysis.

In parallel with these durability signals, safety data from our study showed no statistically significant differences in AEs or SAEs between golimumab and other anti-TNF agents, suggesting a comparable safety and tolerability profile. Although the relatively small number of events makes it difficult to rule out clinically meaningful differences, this observation is consistent with existing evidence supporting the favorable safety of golimumab in routine practice.

Several limitations of this study must be considered when interpreting its results. First, as an observational analysis, it is subject to potential biases related to treatment selection and unmeasured confounders; however, the use of standardized prospective data collection across registries helps lower this risk. Second, the data were drawn from four independent registries, and although data definitions and documentation procedures were harmonized through the UMBRELLA-IBD data warehouse, minor differences in site-level practice or reporting cannot be ruled out; nonetheless, statistical assessment showed minimal heterogeneity, supporting the appropriateness of pooling. Third, while the study reflects routine care in Germany, which may limit generalizability to other health systems, the multicenter nature and inclusion of a broad patient population improve the external validity of the findings in similar healthcare contexts.

Despite these limitations, the use of standardized, prospectively collected registry data across multiple real-world care settings provides a meaningful reflection of clinical practice in Germany. Such data complement evidence from RCTs by capturing treatment patterns, effectiveness, and tolerability among more diverse and representative patient populations.

In conclusion, golimumab represents a safe and effective treatment option for patients with moderately to severely active UC. In addition to the positive findings from pivotal clinical trials, real-world data—including those presented in this analysis—support its clinical effectiveness and safety in routine care. Additionally, observational evidence suggests that golimumab is suitable both for induction and for maintenance of clinical remission. Its subcutaneous administration and infrequent dosing support ease of use and may facilitate long-term adherence. Given the relatively frequent prior use of biologics among our study patients (92%), our data primarily support the use of golimumab in later-line UC patients. The safety profile in this cohort is consistent with previous reports, with no new safety signals identified. Based on current evidence, golimumab can be considered an effective and well-tolerated option for the long-term management of ulcerative colitis. Appropriate patient selection and close clinical monitoring remain essential to optimize therapeutic outcomes.

## Figures and Tables

**Table 1:** Baseline characteristics of patients newly initiating golimumab therapy.

Variable	Value
Number of patients, *n*	222
Age, median (IQR)	39.00 (30.00, 51.40)
Female sex, *n* (%)	110 (49.5)
Time from diagnosis to therapy initiation in years, median (IQR)	5.85 (2.60, 12.80)
Disease extent, *n* (%)	
-E1 (proctitis)	13 (6.1)
-E2 (left-sided colitis)	89 (41.8)
-E3 (extensive colitis)	111 (52.1)
Partial Mayo Score (pMS), median (IQR)	4.00 (2.00, 6.00)
CRP (mg/dL), median (IQR) (data from 157/222 pts.)	2.55 (0.80, 7.84)
Prior exposure to advanced therapies (data from 163/222 pts.)	
-no prior advanced therapies (%)	8.0
-one prior advanced therapy (%)	11
-≥2 prior advanced therapies (%)	81
Body weight in kg, median (IQR)	74.00 (63.00, 86.00)
Patients with body weight ≥ 80 kg, *n* (%)	86 (38.9)

**Table 2:** Golimumab effectiveness through month 12 in patients remaining on golimumab therapy (“as observed” analysis).

	Week 0	Month 6	Month 12
Golimumab patients, *n*	222	138	101
Clinical remission, *n* (%)	33 (15.0)	55 (39.9)	51 (50.5)
Steroid-free clinical remission, *n* (%)	25 (11.4)	50 (36.2)	49 (48.5)

**Table 3:** Remission under golimumab through month 12 in patients <80 kg or ≥80 kg (Data from RUN-UC and VEDO-IBD only).

	Week 0	Weeks 14–16	Month 6	Month 12
<80 kg: *n*	20	18	15	15
Clinical remission, *n* (%)	1 (5.0)	6 (33.3)	5 (33.3)	4 (26.7)
Steroid-free clinical remission, *n* (%)	1 (5.0)	4 (22.2)	4 (26.7)	4 (26.7)
≥80 kg: *n*	16	13	17	13
Clinical remission, *n* (%)	0 (0.0)	2 (15.4)	6 (35.3)	5 (38.5)
Steroid-free clinical remission, *n* (%)	0 (0.0)	2 (15.4)	6 (35.3)	4 (30.8)

None of the differences in remission rates between patients weighing < 80 kg and those ≥80 kg were statistically significant (*p* > 0.05).

## Data Availability

The data underlying this article cannot be shared publicly due to the privacy of the individuals who participated in the study. The data will be shared upon reasonable request made to the corresponding author.

## Supplementary Materials

The following supporting information can be downloaded at: https://www.mdpi.com/article/10.3390/jcm14207347/s1, Figure S1: Golimumab patients flow diagram from the UMBRELLA-IBD study on the effectiveness of golimumab over 12 months (mITT analysis excluding missing outcomes at month 12); Figure S2: 12-month effectiveness of golimumab in ulcerative colitis: comparison of findings from randomized controlled trials (RCTs) and real-world (RW) studies.

## Abbreviations

The following abbreviations are used in this manuscript:

AE	Adverse event
CR	Clinical remission
CRP	C-reactive protein
DGVS	German Society for Gastroenterology, Digestive and Metabolic Disease
ECCO	European Crohn’s and Colitis Organisation
HRQoL	Health-related quality of life
IBD	Inflammatory Bowel Disease
IQR	Interquartile range
mITT	Modified intention-to-treat
pMayo	Partial Mayo Score
RCT	Randomized controlled trial
SAE	Severe adverse event
SFCR	Steroid-free clinical remission
SF-12	12-item short form survey
UC	Ulcerative Colitis
WPAI	Work Productivity and Activity Impairment
