# Supplementary material for: Real-World Effectiveness of Golimumab in Ulcerative Colitis: A Pooled Analysis from the Prospective UMBRELLA-IBD Registry in Germany
Source: J Clin Med. 2025 Oct 17;14(20):7347. doi: 10.3390/jcm14207347 (PMC12565339; doi:10.3390/jcm14207347)
Supplement: Supplementary file 1 [file jcm-14-07347-s001.zip › jcm-3881532-supplementary_updated.pdf]

Supplementary Figures

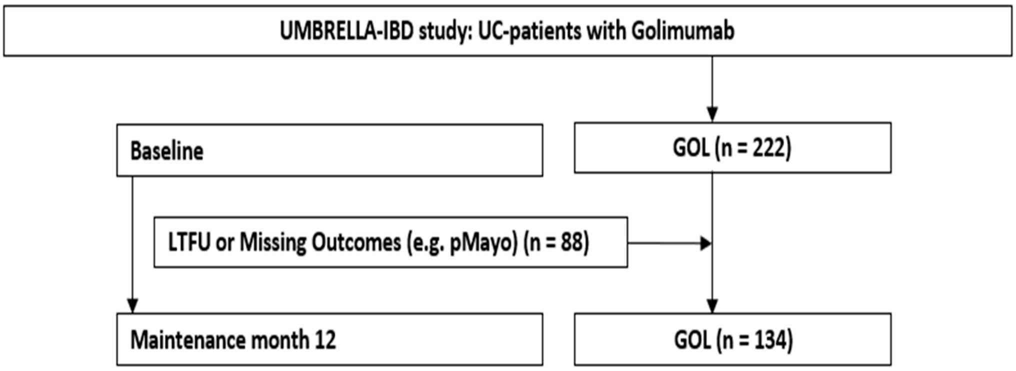

Figure S1. Golimumab patients flow diagram from the UMBRELLA-IBD study on the effectiveness of golimumab over 12 months (mITT analysis excluding missing outcomes at month 12).

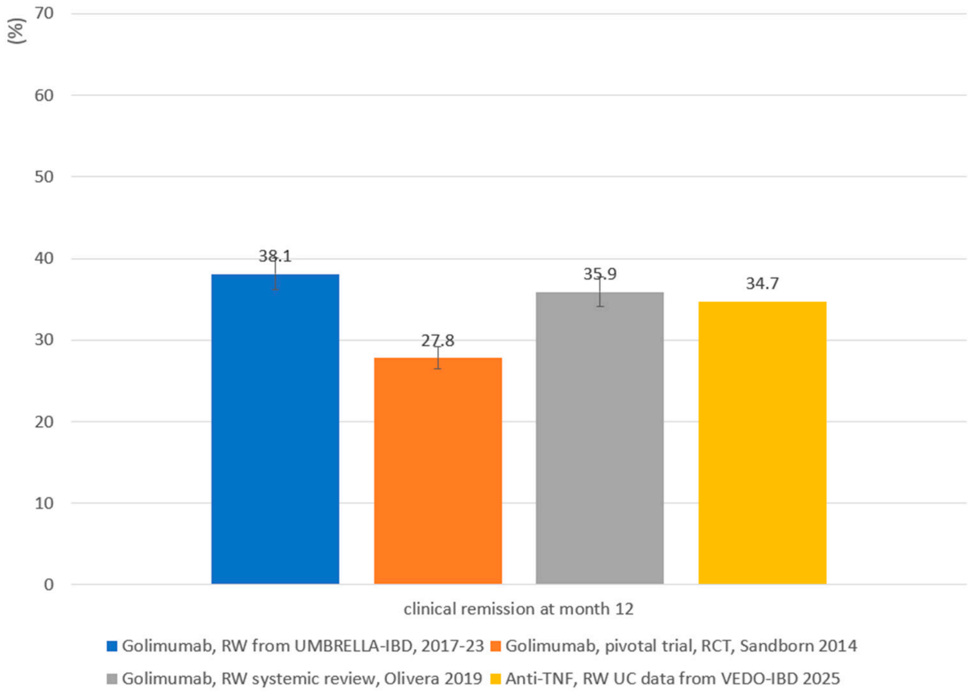

Figure S2. 12-month effectiveness of golimumab in ulcerative colitis: comparison of findings from randomized controlled trials (RCTs) and real-world (RW) studies.
