# Supplementary figures and images for: Real-World Effectiveness of Golimumab in Ulcerative Colitis: A Pooled Analysis from the Prospective UMBRELLA-IBD Registry in Germany
Source: J Clin Med. 2025 Oct 17;14(20):7347. doi: 10.3390/jcm14207347 (PMC12565339; doi:10.3390/jcm14207347)

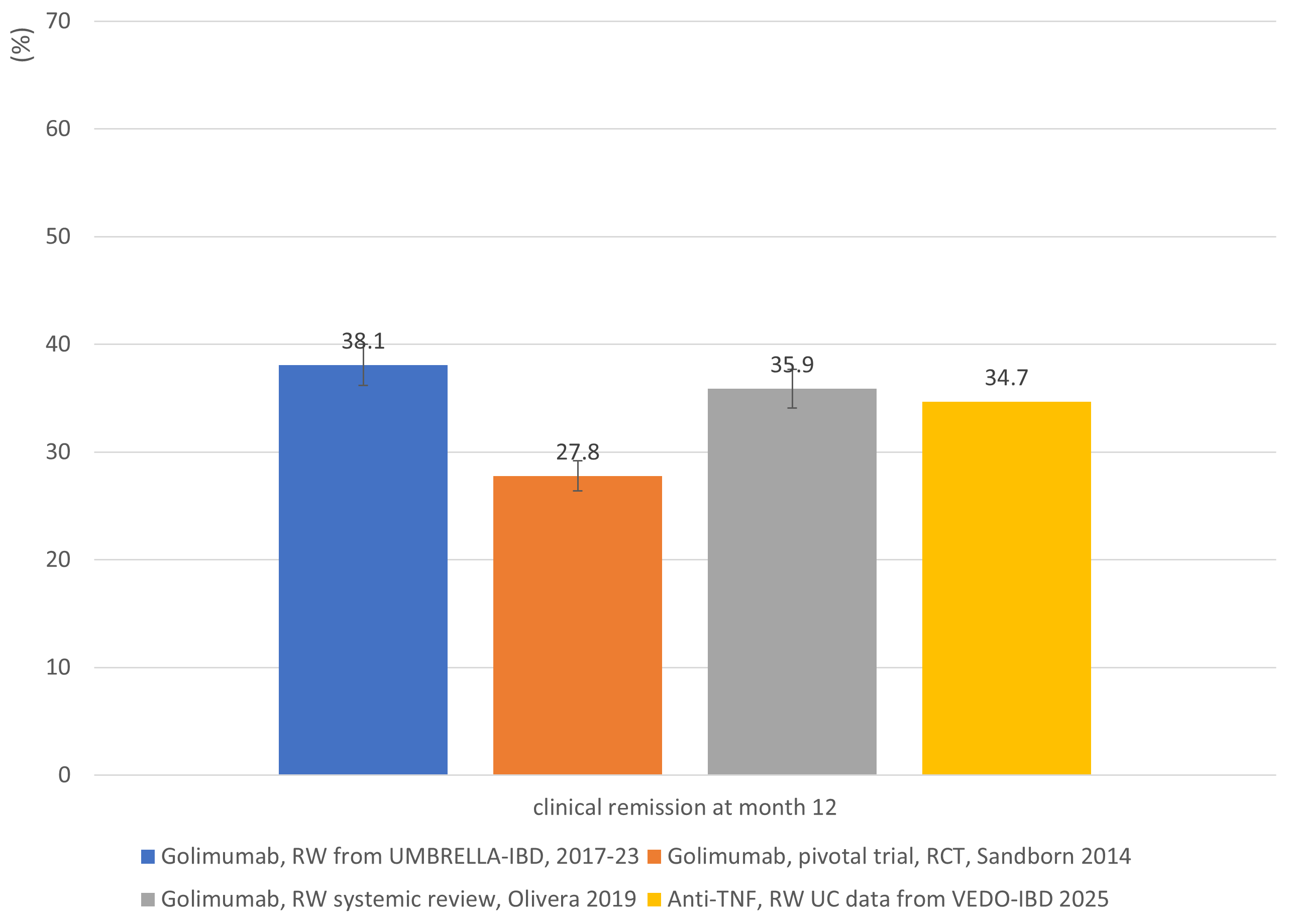

Supplement: Supplementary file 1 [file jcm-14-07347-s001.zip › Supplementary Figure S2.tif]
